# Discovering TEAD4 modulators for hepatocellular carcinoma: a GAN-enabled generative modelling framework

**DOI:** 10.3389/fbinf.2026.1811161

**Published:** 2026-05-04

**Authors:** Varshni Premnath, Ramanathan Karuppasamy, Jayakumar Kaliappan, Shanthi Veerappapillai

**Affiliations:** 1 Department of Biotechnology, School of Bio Sciences and Technology, Vellore Institute of Technology, Vellore, Tamil Nadu, India; 2 Department of Analytics, School of Computer Science and Engineering, Vellore Institute of Technology, Vellore, Tamil Nadu, India

**Keywords:** density functional theory, generative adversarial networks, Hippo-YAP signaling, liver diseases, TEAD4

## Abstract

**Introduction:**

Liver diseases continue to impose a major global health burden, and therapeutic progress is constrained by the limited availability of validated small-molecule modulators. TEAD4, a central Hippo-YAP effector, has emerged as a key regulator of hepatic regeneration, survival, and disease progression, yet remains pharmacologically underexplored due to the scarcity of experimentally confirmed inhibitors. Critically, the limited number of known active compounds restricts effective supervised learning, necessitating data augmentation strategies capable of expanding TEAD4 relevant chemical space.

**Methods:**

To address this, we developed an integrative computational framework in which a conditional generative adversarial network was trained on QikProp-derived molecular descriptors to generate chemically realistic synthetic samples and mitigate class imbalance. This GAN driven augmentation enabled construction of a robust activity prediction model. XGBoost was selected as the classifier due to its strong performance on structured descriptor datasets and its ability to capture complex nonlinear relationships with strong generalization. The augmented dataset was used to train the XGBoost classifier for activity prediction and screen DrugBank compounds, producing a focused set of high confidence candidates. Shortlisted hits were refined using structure-based evaluation, toxicity filtering, and anticancer sensitivity prediction.

**Results:**

Quantum chemical analysis identified DB00169 (cholecalciferol) as a potential TEAD4-binding candidate supported by combined structural, dynamic, and electronic analyses. Molecular dynamics simulations further supported the stability of the TEAD4–ligand complex, indicating compact structural behaviour and thermodynamically favourable conformational states.

**Discussion:**

Overall, this work demonstrates that coupling GAN based molecular augmentation with XGBoost classification and molecular simulations provides a scalable strategy for identifying biologically meaningful TEAD4 modulators, supporting TEAD4 targeted drug discovery across liver diseases.

## 1 Introduction

Liver diseases represent a critical global health challenge, accounting for nearly two million deaths annually, or approximately 4% of all deaths worldwide, with men comprising nearly two-thirds of these fatalities (Devarbhavi et al., 2023). The majority of these deaths result from cirrhosis and hepatocellular carcinoma (HCC), the most common primary liver malignancy. As highlighted by Singh et al. (2025), HCC ranks among the leading causes of cancer-related mortality worldwide. Within this context, TEAD4, a pivotal transcription factor of the Hippo signaling pathway, has emerged as a central regulator of hepatic cell proliferation, differentiation, and tissue homeostasis. Dysregulation of Hippo-TEAD signaling has been implicated not only in malignant transformation but also in abnormal liver regeneration, fibrosis, and inflammatory remodelling. Aberrant activation and overexpression of TEAD4 are therefore increasingly recognized as key molecular events linking chronic liver disease progression to oncogenic transformation, positioning TEAD4 as a therapeutically relevant target across both malignant and non-malignant hepatic disorders (Coto-Llerena et al., 2021).

The Hippo pathway is a core regulator of organ size, tissue homeostasis, and controlled proliferation. When the pathway is inactive, its downstream effectors YAP and TAZ accumulate in the nucleus and bind TEAD transcription factors, activating genes linked to growth, migration, and survival. Dysregulation of this pathway results in sustained nuclear YAP/TAZ activity, driving oncogenic transcriptional programs. In HCC, the YAP-TEAD complex is frequently hyperactivated, promoting aggressive tumor behaviour, metastasis, and resistance to therapy, highlighting the importance of disrupting this interaction to restore proper growth control (Lv and Zhou, 2023).

Nevertheless, effective TEAD4 inhibitors remain elusive. The primary challenges include high structural conservation among TEAD paralogs, the difficulty of achieving nuclear delivery, and the inherent complexity of disrupting protein-protein interactions (Holden and Cunningham, 2018). These limitations underscore the need for advanced, computationally driven strategies to accelerate the discovery and optimization of small molecules capable of selectively targeting TEAD4 with high precision and therapeutic potential.

In this context, machine learning (ML) has rapidly reshaped modern drug discovery by enabling data-driven approaches that reduce experimental time, cost, and uncertainty (Ekins et al., 2019; Vamathevan et al., 2019). By leveraging large-scale molecular datasets, ML models can capture complex nonlinear structure-activity relationships, improving the efficiency of hit identification, lead optimization, and toxicity profiling (Patel et al., 2020). However, for many liver disease relevant targets, experimentally validated bioactivity datasets remain sparse and class-imbalanced, limiting the robustness of conventional supervised modelling and motivating approaches that can enrich the training space. In this regard, generative augmentation strategies, including GAN-based methods, have gained attention and have been increasingly applied in liver-related biomedical settings to overcome small-sample constraints and improve predictive generalization (Mulé et al., 2023).

Among generative approaches, Generative Adversarial Networks (GANs), first introduced by Goodfellow et al. (2014), employ an adversarial training process in which a generator proposes synthetic candidates while a discriminator evaluates their authenticity. Through iterative refinement, GANs learn to emulate the true data distribution, enabling the creation of novel, structurally valid, and pharmacologically meaningful molecular representations (Blanchard et al., 2021). Importantly, this paradigm expands accessible chemical space while directly addressing data scarcity, a persistent bottleneck in early-stage drug discovery (Lin et al., 2020).

Recent studies have demonstrated the versatility of GANs in molecular innovation. Méndez-Lucio et al. (2020) developed a conditional GAN (cGAN) capable of generating molecules aligned with gene-expression signatures, enabling *de novo* hit generation without prior ligand data. Similarly, Blanchard et al. (2021) demonstrated adaptive GAN frameworks that improve molecular diversity and target specificity under small-sample learning conditions. Moreover, Gangwal et al. (2024) provide a comprehensive review of recent GAN architectures tailored for molecular design tasks, including reinforcement learning hybrids and graph-GANs for *de novo* small-molecule generation. However, despite these advancements, applications of GANs to hepatic disorders, particularly HCC, remain sparse. This gap underscores the untapped potential of GAN-driven augmentation for liver disease therapeutics, where data scarcity and structural complexity limit conventional discovery pipelines.

Addressing this gap, the present study introduces the first QikProp descriptor-guided conditional GAN framework for molecule generation targeting key mechanisms in liver diseases. The GAN framework generated 100 additional drug-like molecules within the QikProp descriptor space to expand chemical diversity and improve model learnability. The augmented dataset was subsequently used to train an XGBoost classifier for activity prediction, which was applied to screen the DrugBank database and prioritize candidate therapeutics. Top-ranked hits were then evaluated through molecular docking against TEAD4, establishing an integrated workflow that unites descriptor-informed generative modelling, machine learning–based prioritization, and structure-based validation for accelerated discovery of TEAD4-targeted leads in hepatocellular carcinoma.

**FIGURE 1:**
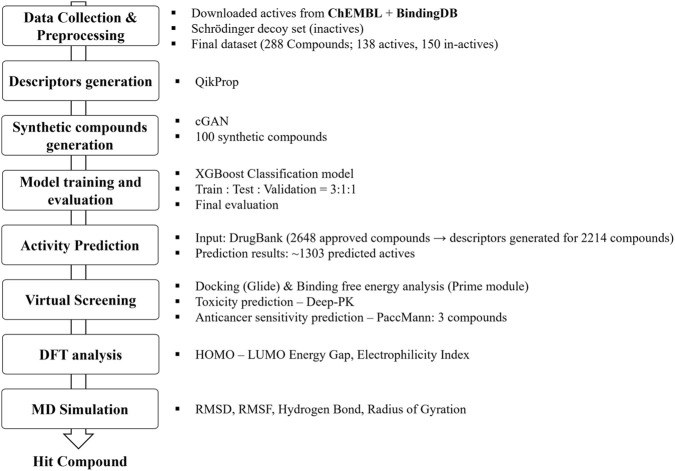
Workflow schematic of the study, illustrating the integration of GAN-based data augmentation, XGBoost modelling, and downstream analysis.

While generative adversarial networks have been widely applied in molecular data augmentation and generation, their utility in descriptor-driven workflows for transcription factor targets such as TEAD4 remains underexplored. In this study, we focus on integrating GAN-based descriptor augmentation with machine learning and multiscale structural analyses to enable systematic prioritization of TEAD4-binding candidates from limited experimental datasets. Figure 1 provides an overview of the workflow adopted in the study.

## 2 Materials and methods

### 2.1 Data collection and descriptors generation

Active compounds were collected from BindingDB (Liu et al., 2025) and ChEMBL (Zdrazil, 2025), filtered based on IC_50_ values. Molecules with IC_50_ ≤ 10,000 nM were retained as actives, while those with higher values were discarded (Das et al., 2024). To ensure balanced datasets for model training, structurally diverse inactive compounds (decoys) were retrieved from Schrödinger’s decoy set. The combined dataset was then standardized by removing duplicates to maintain structural consistency. The QikProp module of Schrödinger, was subsequently employed to calculate molecular descriptors for all active and inactive compounds, encompassing physicochemical, pharmacokinetic, and drug-likeness parameters. These descriptors served as the foundational feature set for both generative modelling and classification model generation.

Physicochemical and pharmacokinetic descriptors were selected as they provide continuous representations of molecular properties relevant to ADME behaviour and drug-likeness. Such descriptors have been shown to be highly informative for machine learning-based prediction tasks and, in certain contexts, can perform comparably or better than traditional fingerprint-based representations, particularly for ADME and property prediction models (Orosz et al., 2022). The final curated dataset consisted of 138 active and 150 inactive compounds prior to augmentation.

### 2.2 Data augmentation using GAN

To address the limited number of experimentally validated compounds and to achieve class balance, a conditional Generative Adversarial Network (cGAN) was implemented using PyTorch (Goodfellow et al., 2014; Mirza and Osindero, 2014). The input descriptor matrix, derived from QikProp, was initially scaled using Min-Max normalization to ensure all features lay within a uniform range. The cGAN consisted of two fully connected neural networks, a generator and a discriminator, trained in an adversarial framework for 1,000 epochs. The generator network comprised three fully connected layers with 128 and 256 neurons in the hidden layers, followed by a sigmoid activation function at the output layer to match the normalized descriptor range. Batch normalization and LeakyReLU activation functions were applied to improve training stability and gradient flow. The discriminator consisted of fully connected layers with 256 and 128 neurons, utilizing LeakyReLU activation, followed by a sigmoid output layer for binary classification. Conditional information was incorporated through label embedding, enabling the generation of class-specific samples corresponding to active and inactive compounds.

The generator received a random noise vector concatenated with class labels (active or inactive) and produced synthetic molecular descriptor vectors, while the discriminator learned to distinguish between real and generated data. To stabilize the adversarial training, label smoothing and fixed random seeds were employed throughout all epochs, helping ensure deterministic and reproducible behaviour (Biswas et al., 2023).

Additional measures, including batch normalization in the generator and controlled training dynamics, were employed to mitigate mode collapse. As the generative model operates on descriptor space rather than explicit chemical structures, validation was performed through statistical comparison of feature distributions rather than structure-based chemical validation.

The model was trained using binary cross-entropy loss and optimized using the Adam optimizer with a learning rate of 1 × 10^−4^ and momentum parameters β_1_ = 0.5 and β_2_ = 0.999. The convergence of both networks was monitored by tracking generator and discriminator losses across epochs, and the resulting training curves were saved for reproducibility. Upon completion, the trained generator produced 100 synthetic molecular descriptors, evenly distributed between active (n = 50) and inactive (n = 50) classes. These generated samples preserved realistic physicochemical distributions as they were derived from the QikProp descriptor space, effectively expanding the chemical diversity available for downstream predictive modelling (Jørgensen et al., 2018; Kingma, 2014).

The cGAN was employed for controlled augmentation rather than large-scale data generation. The limited number of synthetic samples was intentionally chosen to modestly improve class balance and feature coverage while preserving the underlying data distribution and minimizing the risk of overfitting. A fixed random seed (SEED = 42) was applied consistently across all stages of the workflow, including GAN training, synthetic data generation, dataset splitting, and XGBoost model training, to ensure deterministic and reproducible results.

To evaluate the quality of the generated samples, statistical validation was performed using dimensionality reduction techniques, including principal component analysis (PCA) and t-distributed stochastic neighbour embedding (t-SNE). PCA was performed to capture the principal variance in the descriptor space, while t-SNE was applied with a perplexity of 30 and 1,000 iterations to preserve local neighbourhood structure. Additionally, distributional comparisons of selected molecular descriptors were conducted between real and synthetic datasets to assess consistency in physicochemical feature space.

### 2.3 Classification model training

To ensure unbiased evaluation, the dataset was first split into training, validation, and test subsets using only real compounds. Synthetic samples generated by the cGAN were subsequently incorporated exclusively into the training set to enhance feature representation, while validation and test sets contained only real data. The training data, including both real and synthetic samples, were then standardized using z-score normalization to ensure uniform feature scaling prior to model training (Henderi et al., 2021). This strategy prevents data leakage and ensures a realistic assessment of model generalization on experimentally derived compounds.

A gradient boosting algorithm, implemented through the XGBoost framework, was then used to classify active and inactive molecules based on their descriptor profiles. The model was configured with 2,000 estimators, a learning rate of 0.03, a maximum tree depth of 6, subsampling of 0.9, and column sampling of 0.8 per tree to balance bias and variance. Regularization parameters (λ = 1.0, α = 0.0) were included to prevent overfitting, and a fixed random seed ensured reproducibility. Model training employed the “hist” tree-building method and was evaluated using log-loss as the metric for both training and validation datasets. The learning curves of log-loss across boosting rounds were analyzed to confirm convergence, and the final model was exported for deployment and reproducibility (Chen, 2016). No explicit feature selection was performed, and the complete set of QikProp descriptors was used to retain the full physicochemical representation of the compounds.

### 2.4 Model evaluation

The performance of the trained XGBoost classifier was rigorously assessed using an independent test subset that was withheld during training to ensure an unbiased evaluation of generalization. Standard classification metrics were computed to quantify predictive performance, providing a comprehensive view of both sensitivity and specificity. In addition, the confusion matrix was examined to identify the distribution of true positives, true negatives, false positives, and false negatives, offering deeper insight into model behaviour across both classes (Powers, 2020).

### 2.5 Activity prediction on external dataset

To evaluate the model’s applicability to unseen compounds, the trained XGBoost classifier was applied to an external dataset derived from DrugBank. QikProp descriptors for these compounds were pre-computed and processed using the same normalization and standardization parameters applied to the training data, ensuring consistent feature scaling (Vamathevan et al., 2019). The model predicted activity probabilities for each compound, which were subsequently thresholded at 0.5 to classify them as either active or inactive. The resulting probabilities and class predictions were appended to the dataset and exported for downstream analyses. Top-ranked compounds, identified by their high predicted activity scores, were selected for molecular docking against TEAD4 to validate their binding potential.

### 2.6 Protein and ligands preparation

The 3D crystal structure of TEAD4 (PDB ID: 8CAA) (Sellner et al., 2023) was obtained from the Protein Data Bank (Berman et al., 2000) and processed using the Protein Preparation Wizard module in Maestro. The workflow included preprocessing, optimization, and minimization. During preprocessing, missing hydrogen atoms were added, bond orders were assigned, disulfide linkages were generated, and water molecules beyond 5 Å from the binding site were removed (Madhavi Sastry et al., 2013). The final energy minimization was performed under the OPLS3e force field to relieve steric clashes and optimize local geometry (Jorgensen and Tirado-Rives, 1988).

Ligand preparation was performed using the LigPrep module. The reference inhibitor, IAG933 (Chapeau et al., 2024), was downloaded directly from the PubChem database (Kim et al., 2023) and predicted active compounds were obtained from the DrugBank database in SDF format and imported into Maestro for preparation. Ionization states were generated at pH 7.0 ± 0.5 using Epik (Shelley et al., 2007), and low-energy stereoisomers were retained. All ligands were minimized under the OPLS3e force field to ensure geometry and energy consistency before docking. The known TEAD4 inhibitor IAG933 was included as a reference compound to enable retrospective benchmarking of ligand binding behaviour and to support comparative evaluation within the computational workflow.

### 2.7 Molecular docking

Prepared ligands were docked into the refined TEAD4 receptor using the Glide module of the Schrödinger Suite (Halgren et al., 2004). The receptor grid was generated around the ligand-binding site, defined by key interacting residues–Q269, K273, K297, S388, E391, T394, and E416 (Sellner et al., 2023). Docking was performed in a hierarchical manner, beginning with High-Throughput Virtual Screening (HTVS) to eliminate low-affinity ligands, followed by Standard Precision (SP) and finally Extra Precision (XP) docking to obtain high-confidence binding poses and accurate scoring.

### 2.8 MM-GBSA binding free energy calculations

While Glide XP docking offers a robust initial ranking of ligand affinities, its scoring function does not fully account for detailed energetic contributions. To achieve a more reliable estimate of ΔG_bind_, the XP pose-viewer outputs were subsequently evaluated using the Prime MM-GBSA framework (Lyne et al., 2006). The calculations were performed with the OPLS3e force field in conjunction with the Generalized Born Surface Area (GBSA) continuum solvent model. Each docked complex underwent local optimization in Prime to relax side-chain conformations and eliminate steric clashes prior to energy evaluation (Beard et al., 2013; Hou et al., 2011).

The binding free energy (ΔG_bind_) of each ligand–protein complex was calculated as:

ΔGbind=Ecomplex minimized – Eligand minimized+Ereceptor minimized

where, E_complex (minimized)_, E_receptor (minimized)_, and E_ligand (minimized)_ represent the minimized energies of the complex, the isolated receptor, and the unbound ligand, respectively. These minimized energies incorporate contributions from molecular mechanics terms, solvation effects, and surface-area interactions, collectively capturing the energetic favourability of ligand binding (Saranyadevi and Shanthi, 2020).

### 2.9 Toxicity prediction and anticancer sensitivity prediction

The pharmacokinetic and toxicological profiles of the docked ligands were assessed using Deep-PK, a deep-learning platform that predicts multiple ADMET endpoints. Toxicity evaluations included Ames mutagenicity, carcinogenic potential, and drug-induced liver injury (Myung et al., 2024). Predicted non-toxic molecules were further analyzed for anticancer potential using PaccMann, a transcriptomics-driven neural network model that estimates cytotoxic activity across cancer cell lines (Cadow et al., 2020). The evaluation focused on two common hepatic carcinoma lines - HEPG2 and HUH7 to identify compounds likely to exhibit selective anti-HCC activity (Nwosu et al., 2018).

### 2.10 Density functional theory (DFT)

Electronic structure calculations were carried out using DFT within the Jaguar module of the Schrödinger Suite to explore quantum-level reactivity descriptors of the top-ranked ligands (Bochevarov et al., 2013). The lowest-energy conformations from molecular docking were subjected to geometry optimization followed by single-point energy calculations at the B3LYP/6-31G** level of theory. Frontier molecular orbitals–HOMO (highest occupied molecular orbital) and LUMO (lowest unoccupied molecular orbital) were analyzed to determine electron donating and accepting tendencies, respectively, and their energy difference (ΔE_HL_ = E_LUMO_ − E_HOMO_) was used to infer chemical stability and reactivity (Krishnamoorthy and Karuppasamy, 2023).

Global reactivity descriptors, including electronegativity (χ), chemical potential (μ), hardness (η), softness (S), and electrophilicity index (ω), were computed to characterize ligand behaviour at the ground state. Ionization potential (I) and electron affinity (A) were derived following Koopmans’ theorem, with

$$I=-E_{\text{HOMO}},A=-E_{\text{LUMO}}$$

and subsequent descriptors calculated as:

$$\eta =\frac{\left(I-A\right)}{2},\;S=\frac{1}{\eta },\;\chi =\frac{\left(I+A\right)}{2},\;\mu =-\chi ,\;\omega =\frac{\mu^{2}}{2\times \eta }$$

These DFT parameters provide insights into molecular stability, charge transfer, and binding reactivity, thereby supporting the interpretation of physicochemical behaviour and biological potential of the candidate inhibitors (Parr et al., 1999; Pearson, 1988).

### 2.11 Molecular dynamics simulations

The dynamic stability and conformational behaviour of the TEAD4-ligand complexes were investigated through molecular dynamics (MD) simulations using the GROMACS 2020.3 software package (Van Der Spoel et al., 2005). The missing internal loop segments in the TEAD4 structure were reconstructed during protein preparation using the Protein Preparation Wizard in Maestro to ensure a continuous and physically realistic protein structure prior to simulation. This reconstruction ensured a continuous protein backbone suitable for molecular dynamics simulations. Topology parameters for the selected docked ligands were generated using the CGenFF server, while the protein topology was prepared with the CHARMM36 force field (Vanommeslaeghe et al., 2010). Each complex was solvated explicitly within a dodecahedral simulation box using the Simple Point Charge (SPC) water model, and the systems were neutralized by adding sodium counterions as required (Murali and Karuppasamy, 2023).

Energy minimization was performed ensuring removal of steric clashes. Equilibration was conducted in two successive stages: an NVT (constant number, volume, temperature) ensemble followed by an NPT (constant number, pressure, temperature) ensemble, each for 100 ps. The temperature and pressure were maintained at 300 K and 1 bar using the Berendsen thermostat and Parrinello - Rahman barostat, respectively. Following equilibration, a 100 ns MD simulation was executed under periodic boundary conditions to capture long-timescale conformational dynamics. The trajectory data were analyzed to evaluate the root mean square deviation (RMSD) and root mean square fluctuation (RMSF) of the protein backbone, hydrogen-bond occupancy, radius of gyration (Rg), solvent-accessible surface area (SASA), and free-energy landscape (FEL) profiles. All trajectory analyses were carried out using built-in GROMACS utilities, providing insights into complex stability, compactness, and conformational adaptability during the simulation period (Thirunavukkarasu et al., 2021).

## 3 Results

### 3.1 GAN-based data augmentation

**FIGURE 2:**
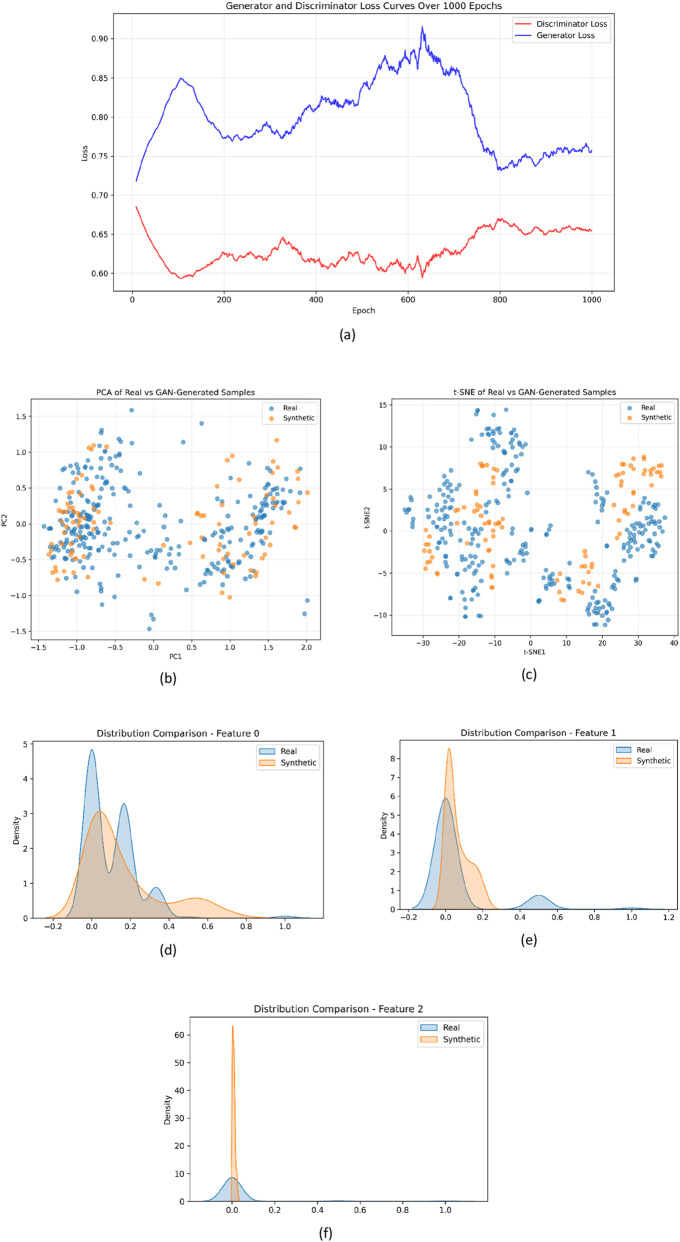
**(a)** Generator and discriminator loss curves during cGAN training, showing the stabilization of adversarial learning over 1,000 epochs, **(b)** Principal component analysis (PCA) projection of real and synthetic samples, demonstrating substantial overlap in reduced-dimensional descriptor space, **(c)** t-distributed stochastic neighbour embedding (t-SNE) visualization of real and synthetic samples, confirming preservation of local data structure and absence of mode collapse (real samples are shown in blue, while synthetic samples are shown in orange) **(d–f)** Distributional comparison of representative molecular descriptors between real and synthetic datasets, illustrating consistency in physicochemical feature distributions.

The cGAN was successfully trained to generate synthetic molecular descriptors representative of both active and inactive compounds. The adversarial training dynamics are illustrated in Figure 2a, where the generator and discriminator loss curves demonstrate progressive stabilization after approximately 700 epochs, indicating the attainment of equilibrium between the two competing networks. Early epochs were characterized by oscillations in loss values, reflecting the generator’s gradual adaptation to the discriminator’s evolving feedback. The discriminator initially exhibited lower loss values, suggesting its dominance in identifying synthetic samples. However, with continued iterations, the generator improved its ability to reproduce realistic descriptor distributions, as evidenced by the convergence of both loss profiles.

The discriminator loss plateauing near 0.65 and generator loss stabilizing around 0.75 signify that neither network overpowered the other, confirming a balanced adversarial state. This equilibrium suggests that the generator successfully captured the complex feature correlations underlying the QikProp descriptor space without overfitting or mode collapse. The resulting synthetic data displayed realistic variability across lipophilicity, hydrogen-bonding capacity, and surface polarity, mirroring the trends seen in the original dataset.

To further assess the quality and distributional fidelity of the generated samples, dimensionality reduction analyses were applied. As shown in Figure 2b, the principal component analysis projection reveals substantial overlap between real (blue) and synthetic (orange) samples within the reduced feature space. The absence of distinct clustering between the two groups indicates that the generated descriptors closely follow the global variance structure of the original dataset, suggesting that the cGAN effectively learned the underlying feature distribution rather than producing out-of-distribution artifacts.

Complementary to this, the t-distributed stochastic neighbour embedding visualization (Figure 2c) provides insight into the preservation of local data structure. The intermixing of real (blue) and synthetic (orange) samples across multiple regions of the embedding space demonstrates that the generated data not only align with global trends but also capture fine-grained neighbourhood relationships. Importantly, the absence of dense, isolated clusters of synthetic samples indicates that mode collapse did not occur, and that the generator maintained diversity across the descriptor space.

In addition to spatial distribution analyses, feature-level validation was performed through direct comparison of representative molecular descriptor distributions (Figures 2d–f), including key descriptors such as logP, polar surface area, and hydrogen-bond donor/acceptor counts. The generated samples exhibit strong concordance with the real dataset across key physicochemical properties, including lipophilicity, polarity, and hydrogen-bonding capacity. While minor deviations are observed in certain descriptor ranges, these variations reflect the model’s ability to generalize dominant data patterns rather than replicate individual data points, thereby contributing to controlled diversity without distorting the overall distribution.

Collectively, these statistical validations confirm that the cGAN-generated descriptors preserve both global and local structural characteristics of the original dataset while introducing meaningful variability. This supports their suitability for downstream machine learning applications and reinforces the robustness of the augmentation strategy employed in this study.

The generation of 100 balanced synthetic samples provided improved representation across classes and modest expansion of the descriptor space for downstream classification. This controlled augmentation strategy enhanced feature diversity while maintaining the underlying data distribution, which is particularly important given the relatively balanced nature of the original dataset. Collectively, these analyses provide statistical validation of the quality, diversity, and distributional consistency of the generated samples.

### 3.2 Performance of the XGBoost classification model

**FIGURE 3:**
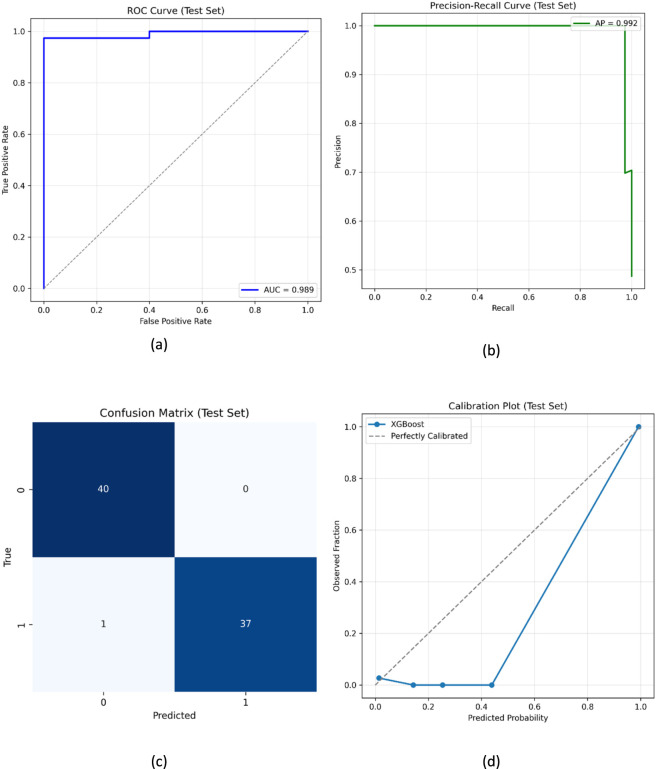
Performance evaluation of the XGBoost model trained on GAN-augmented data **(a)** ROC curve on the test set demonstrating excellent discrimination between active and inactive compounds (AUC = 0.99) **(b)** Precision-Recall curve highlighting high precision and recall for both classes (average precision = 0.99) **(c)** Confusion matrix **(d)** Calibration plot indicating that predicted probabilities closely match observed frequencies.

The augmented dataset was subsequently used to train an XGBoost classifier, which demonstrated excellent predictive capability in distinguishing active from inactive compounds. The Receiver Operating Characteristic (ROC) curve shown in Figure 3a exhibited an Area Under the Curve (AUC) of 0.989, indicating excellent discrimination performance. The classifier maintained high sensitivity and specificity across a broad range of probability thresholds, demonstrating its robustness even under varying decision boundaries. The Precision-Recall (PR) curve (Figure 3b) further confirmed this observation, with an average precision exceeding 0.992, underscoring the model’s reliability in identifying true actives within an imbalanced chemical landscape.

The confusion matrix (Figure 3c) depicted an almost symmetric distribution of true positives and true negatives, indicating balanced classification across both classes. Misclassifications were minimal, suggesting that the GAN-based augmentation contributed effectively to feature diversity and prevented bias toward the majority class. Furthermore, the probability calibration curve (Figure 3d) displayed a nearly linear alignment between predicted probabilities and observed frequencies, validating that the model’s output probabilities are well-calibrated and interpretable for real-world screening scenarios.

**FIGURE 4:**
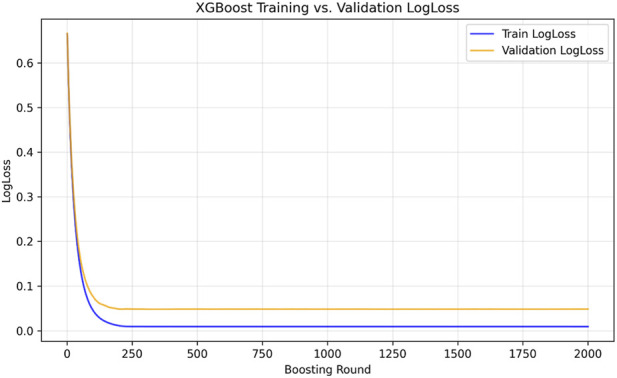
Training and validation logloss curves during XGBoost training on GAN-augmented data. The training logloss decreases steadily, while the validation logloss stabilizes after approximately 500 boosting rounds, indicating stable convergence and minimal overfitting. This figure demonstrates that the model generalizes well and that additional boosting rounds do not significantly improve validation performance.

The training dynamics of XGBoost, illustrated in Figure 4, provided further insight into the learning behaviour. The training log-loss curve showed a steady decline over the boosting iterations, plateauing near convergence, while the validation log-loss followed a similar trend with only minimal deviation, indicating the absence of overfitting. The synchronized convergence of training and validation losses after approximately 500 boosting rounds confirms the model’s stability and its ability to capture meaningful nonlinear relationships within the descriptor space.

Importantly, the model’s deterministic design achieved by fixed random seeds, single-threaded computation, and GAN-augmented balance ensured reproducibility across runs, a critical requirement for robust AI-driven drug discovery. Collectively, these results demonstrate that the combination of descriptor-level GAN augmentation and XGBoost learning forms a synergistic framework capable of precise activity classification and confident probability estimation for TEAD4 modulator prediction.

In addition to graphical evaluation, quantitative performance metrics were computed to provide a comprehensive assessment of model performance. On the independent test set, the model achieved an accuracy of 0.987, precision of 1.000, reflecting the absence of false positives in the test set, recall of 0.974, F1-score of 0.987, ROC-AUC of 0.989, and Matthews correlation coefficient (MCC) of 0.975, indicating strong and well-balanced predictive performance across both classes. The high precision indicates minimal false positive predictions, while the strong recall reflects effective identification of active compounds. The elevated MCC value further confirms consistent classification performance without class bias. To further evaluate robustness, stratified 5-fold cross-validation was performed on the training dataset. The model achieved a mean accuracy of 0.983 with a standard deviation of 0.016, demonstrating consistent performance across folds and supporting its generalizability.

### 3.3 Prediction of potential TEAD4 modulators

The trained XGBoost classification model was employed to screen approved compounds subset from DrugBank database to identify potential TEAD4 binding candidates. A total of 2,648 compounds were initially retrieved, of which 2,214 successfully yielded QikProp descriptors compatible with the model’s feature set. These processed descriptors were normalized using the same scaling parameters applied during model training to maintain feature consistency. The model predicted 1,303 compounds as active, representing approximately 58% of the screened dataset.

### 3.4 Docking and binding free energy calculations

The shortlisted compounds predicted as active were docked into the prepared TEAD4 receptor using the optimized grid defined by key residues Q269, K273, K297, S388, E391, T394, and E416. A hierarchical docking protocol was used to obtain refined ligand orientations and interaction profiles. The docking study primarily served as an initial structural screening step to identify plausible binding poses. To obtain a more reliable estimate of binding affinity, the analysis was further refined using the Prime MM-GBSA approach, which has been reported to provide improved correlation with experimental binding data compared to docking scores alone (Gen heden and Ryde, 2015; Hou et al., 2011).

Post-docking rescoring was carried out using the Prime MM-GBSA method with the OPLS3e force field. The reference ligand, IAG933, was also subjected to identical calculations to serve as a benchmark for binding affinity comparison. This inclusion provides a reference-based comparative framework, allowing direct comparison between a known TEAD4 inhibitor and the predicted candidates in terms of binding affinity and interaction stability. The resulting MM-GBSA energies provided quantitative insights into the relative stability of the predicted ligand complexes. Table 1 summarizes the binding free-energy values for the top-scoring compounds, representing the most favourable subset from the DrugBank screen. Several predicted candidates exhibited comparable or more favourable ΔG_bind_ values relative to the reference compound, suggesting potential for stable binding within the TEAD4 pocket. These computational estimates are intended for relative comparison of binding potential and should not be interpreted as definitive indicators of biological activity.

**TABLE 1:** Predicted binding free energies of TEAD4 - ligand complexes.

Compound	dG bind (kcal/mol)	Coulomb energy (kcal/mol)	Covalent interaction (kcal/mol)	Hydrogen bond energy (kcal/mol)	Lipophilicity (kcal/mol)	Solvation energy (kcal/mol)	van der waals energy (kcal/mol)	Ligand strain energy (kcal/mol)
IAG933	**−27.63**	**−18.46**	**0.12**	**−1.56**	**−11.9**	**30.84**	**−25.15**	**3.159928**
DB11936	−43.2	−27.17	4.35	−1.9	−8.59	22.26	−32.16	4.589397
DB00146	−37.4	−8.71	2.73	−1.06	−17.95	18.66	−31.07	2.971653
DB12554	−34	−12.46	−0.12	−1.5	−15.9	27.98	−31.43	3.326699
DB08834	−31.96	−30.79	6.57	−2.96	−11.37	39.81	−33.22	7.221094
DB14477	−31.2	−4.71	4.3	−0.22	−15.11	21.05	−36.49	5.68547
DB00169	−31.13	−0.41	1.64	−0.52	−17.73	16.84	−30.96	2.03289
DB00163	−29.04	−8.47	3.49	−1.15	−13.83	23	−32.04	2.541039

*All binding free energy values are expressed in kcal/mol.

Bold values indicate the reference compound IAG933 used for comparative analysis.

### 3.5 Toxicity and anticancer sensitivity prediction

All compounds that exhibited favourable binding free energies from the Prime MM-GBSA analysis were subjected to toxicity evaluation using the Deep-PK platform. The analysis covered multiple toxicity endpoints, including Ames mutagenicity, carcinogenicity, and drug-induced liver injury (DILI). As summarized in Table 2, all screened compounds displayed acceptable safety profiles when compared with the reference compound IAG933, which showed predicted hepatotoxicity in the DILI category. In contrast, all newly identified ligands were classified as non-toxic and thus considered safe for subsequent evaluation.

**TABLE 2:** Toxicity profiles of selected lead compounds assessed by Deep-PK.

Compound	Ames toxicity	Carcinogenesis	Liver injury I	Liver injury II
IAG933	**0.012 (safe)**	**0.008 (safe)**	**0.252 (safe)**	**0.842 (toxic)**
DB11936	0 (safe)	0.188 (safe)	0.278 (safe)	0.414 (safe)
DB00146	0.047 (safe)	0.411 (safe)	0.244 (safe)	0.489 (safe)
DB12554	0.04 (safe)	0.152 (safe)	0.361 (safe)	0.48 (safe)
DB08834	0.081 (safe)	0.067 (safe)	0.403 (safe)	0.497 (safe)
DB14477	0 (safe)	0.139 (safe)	0.415 (safe)	0.457 (safe)
DB00169	0.06 (safe)	0.335 (safe)	0.288 (safe)	0.449 (safe)
DB00163	0 (safe)	0.27 (safe)	0.349 (safe)	0.435(Safe)

Bold values indicate the reference compound IAG933 used for comparative analysis.

These non-toxic compounds were next analyzed for their anticancer sensitivity against hepatocellular carcinoma (HCC) cell lines using transcriptomic-based prediction models. The results indicated that several of the screened molecules possessed strong predicted activity in hepatic cancer cell lines, suggesting their potential as novel TEAD4-targeted therapeutic agents. The compounds exhibiting both favourable toxicity profiles and predicted anticancer sensitivity are summarized in Table 3 and were shortlisted for advanced electronic-structure and dynamic analyses.

**TABLE 3:** Anticancer activity of selected compounds against liver cancer cell lines predicted by PaccMann.

Compound	HEPG2	HUH7
IAG933	**2.834881111**	**4.46379812**
DB11936	3.205922883	6.566624988
DB00146	0.463013068	1.562051467
DB12554	2.549761945	4.982839952
DB08834	0.356650132	0.70468809
DB14477	2.060605764	5.989452466
DB00169	0.749012205	3.205922883
DB00163	1.95228406	6.079971449

Bold values indicate the reference compound IAG933 used for comparative analysis.

### 3.6 Density functional theory

The top-ranked ligands–Calcifediol (DB00146), Tauroursodeoxycholic acid (DB08834), and Cholecalciferol (DB00169) were subjected to DFT analysis to evaluate their electronic characteristics and stability. The reference compound IAG933 was included for comparative assessment to understand relative reactivity and stability profiles. The computed quantum chemical descriptors are summarized in Table 4.

**TABLE 4:** Calculated quantum chemical descriptors of the lead molecules.

Compound	HOMO	LUMO	Energy gap	I^a^	A^b^	η^c^	σ^d^	χ^e^	μ^f^	ω^g^
IAG933	**−0.210331**	**−0.042164**	**0.168167**	**0.210331**	**0.042164**	**0.0840835**	**11.89293976**	**0.1262475**	**−0.1262475**	**0.00067008**
DB00146	−0.201029	−0.025666	0.175363	0.201029	0.025666	0.0876815	11.40491438	0.1133475	−0.1133475	0.000563251
DB08834	−0.20153	−0.026419	0.175111	0.20153	0.026419	0.0875555	11.42132704	0.1139745	−0.1139745	0.000568681
DB00169	−2.0153	−0.016585	1.998715	2.0153	0.016585	0.9993575	1.000642913	1.0159425	−1.0159425	0.515738007

Bold values indicate the reference compound IAG933 used for comparative analysis.

All parameters are represented in eV.

I^a^- Ionization potential, A^b^–Electron affinity, η^c^–Hardness, σ^d^–Softness, χ^e^–Electronegativity, μ^f^–Chemical potential, ω^g^–Electrophilicity index.

The analysis focused on the frontier molecular orbitals, HOMO (Highest Occupied Molecular Orbital) and LUMO (Lowest Unoccupied Molecular Orbital), whose energy difference (ΔE = E_LUMO_–E_HOMO_) determines a compound’s chemical reactivity and kinetic stability. A larger HOMO–LUMO gap typically corresponds to a more stable and less reactive molecule, while a smaller gap reflects higher reactivity and lower thermodynamic stability.

Among the screened compounds, Cholecalciferol (DB00169) exhibited the highest energy gap of about 1.99 eV, compared to the other ligands and the reference compound, indicating superior electronic stability and a lower tendency for spontaneous excitation or decomposition. This balanced energy profile suggests that DB00169 can maintain structural stability and appropriate electronic properties that may support molecular interactions, when interpreted alongside structural and dynamic analyses. Furthermore, DB00169 showed moderate hardness and high softness, indicating optimal flexibility in adapting to the electrostatic environment of the binding pocket. Its chemical potential and electrophilicity index further support its efficient charge-transfer and stabilization capability upon complex formation.

Overall, the DFT findings reveal that DB00169 (Cholecalciferol) exhibits comparatively favourable quantum chemical characteristics–greater electronic stability, balanced reactivity, and efficient charge distribution compared with both the reference and other shortlisted ligands. This electronic behaviour supports its intrinsic stability and reactivity profile, complementing the binding and dynamic analyses rather than directly indicating biological activity. It is important to note that DFT-derived descriptors were used to complement structural and molecular dynamics analyses and were not employed as standalone indicators of binding affinity or biological activity.

### 3.7 Interaction profiling of TEAD4-Ligand complexes

**FIGURE 5:**
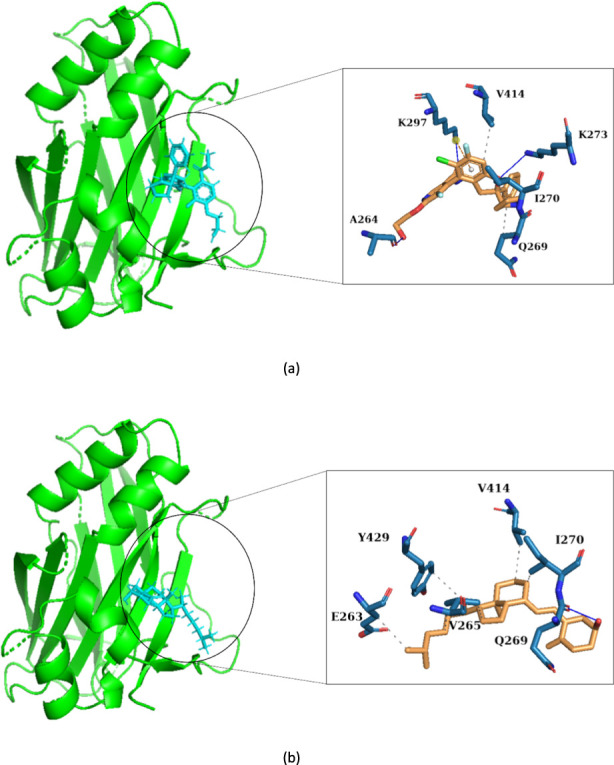
Interaction profiles of the identified hit molecules with TEAD4, highlighting key binding interactions and residues **(a)** Reference - IAG933 **(b)** DB00169.

To dissect the molecular determinants governing ligand recognition by TEAD4, protein–ligand interaction profiles of both the reference compound and the top-hit molecule (DB00169) were characterized using PLIP (Protein–Ligand Interaction Profiler). The interaction maps presented in Figure 5 illustrate the precise binding orientation and residue level contacts stabilizing each complex.

For IAG933, PLIP identified a combination of hydrophobic and hydrogen-bonding interactions that collectively anchor the ligand within the TEAD4 pocket. Three hydrophobic contacts were formed with residues Q269, I270, and V414, reflecting the compound’s ability to engage the non-polar cavity that defines the TEAD4 binding site. Additionally, three hydrogen bonds were detected, one each with A264, K273, and K297. These interactions involve donor–acceptor distances ranging from 2.03 to 2.78 Å, consistent with stable, directional hydrogen bonding. A single π–cation interaction with K297 further contributes to pocket stabilization through aromatic–electrostatic complementarity.

In contrast, DB00169 exhibited a more hydrophobic-rich binding pattern, forming six hydrophobic contacts with residues E263, V265, I270, V414, and Y429, indicating deeper and more extended engagement across the lipophilic groove of TEAD4. Hydrogen bonding was more limited but included a stable interaction with Q269 (H–A distance 2.02 Å), which anchors the molecule in a similar orientation to the reference compound. Overall, PLIP analysis revealed that while the reference compound relies on a balance of hydrogen bonds and electrostatic contacts, DB00169 achieves comparable stabilization primarily through an expanded hydrophobic footprint and conserved polar anchoring at Q269.

Importantly, several of the residues engaged by both IAG933 and DB00169, particularly Q269, K273, K297, and V414, have been previously identified as critical components of the YAP–TEAD interaction surface. Prior structural studies have shown that these residues contribute directly to the hydrophobic and polar interfaces required for YAP anchoring; therefore, ligand occupation at this site is expected to sterically and energetically disrupt YAP binding (Chapeau et al., 2024; Sellner et al., 2023; Zhao et al., 2023).

### 3.8 Molecular dynamics simulations

To assess the conformational stability and dynamic behaviour of the TEAD4–ligand complexes, 100 ns molecular dynamics (MD) simulations were performed for both the reference compound IAG933 and the lead hit DB00169 (Cholecalciferol). Multiple structural parameters, including root mean square deviation (RMSD), root mean square fluctuation (RMSF), hydrogen bond dynamics, radius of gyration (Rg), solvent-accessible surface area (SASA), and Gibbs free energy landscapes (FEL), were analyzed to characterize complex stability and conformational adaptability during the trajectory.

#### 3.8.1 Root mean square deviation (RMSD)

**FIGURE 6:**
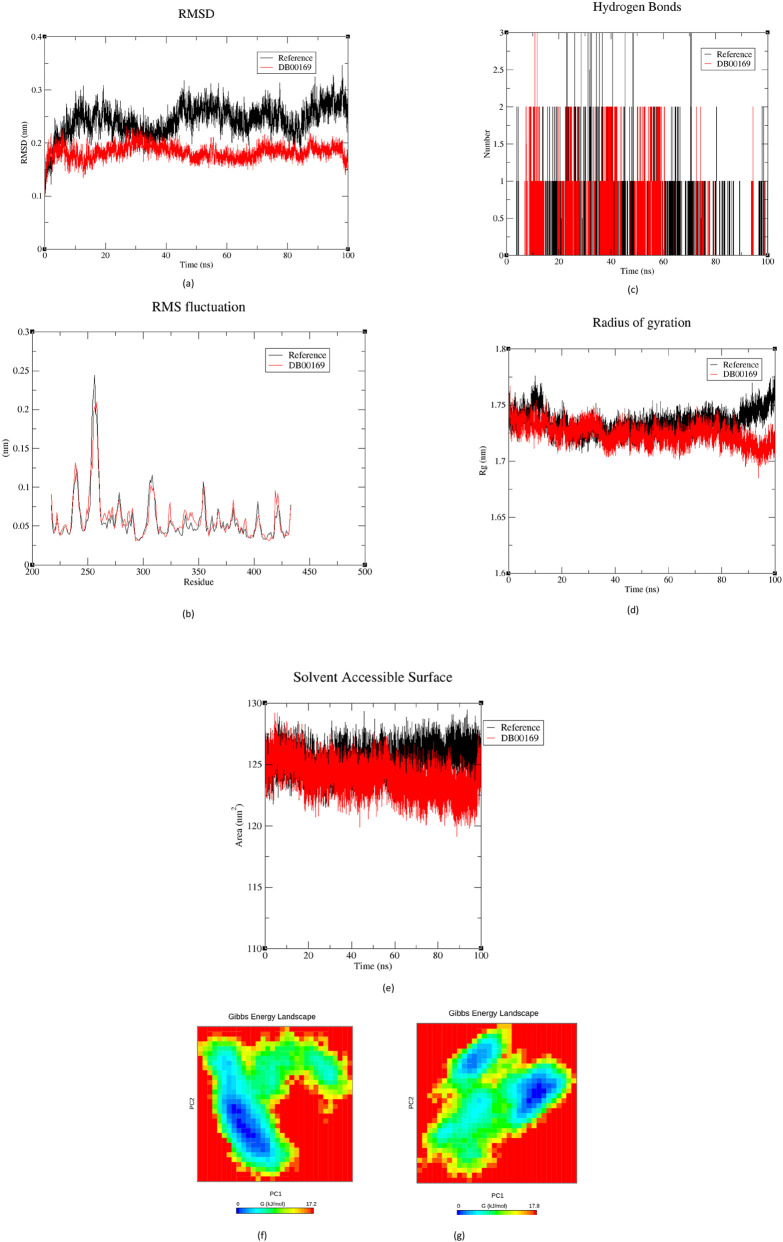
Molecular dynamics simulation profiles of TEAD4 complexes with the reference ligand and the top-hit compound: **(a)** root mean square deviation (RMSD), **(b)** root mean square fluctuation (RMSF), **(c)** hydrogen-bond occupancy, **(d)** radius of gyration (Rg), **(e)** solvent-accessible surface area (SASA), and **(f)** free-energy landscape (FEL) of IAG933 **(g)** FEL of DB00169.

The backbone RMSD trajectories for the TEAD4 - ligand complexes over 100 ns are presented in Figure 6a. Both simulations showed an initial equilibration phase within the first 10 ns, after which the RMSD values stabilized, indicating that the protein had reached a dynamically steady state. For the reference complex, RMSD values predominantly fluctuated between 0.25 and 0.35 nm, with small intermittent rises. These fluctuations reflect minor adjustments within the protein’s global conformation but remain well within the expected range for a stable complex.

In contrast, the TEAD4 - DB00169 system exhibited slightly lower RMSD values, typically between 0.15 and 0.22 nm following equilibration. The absence of higher-amplitude deviations indicates that the ligand anchors TEAD4 in a compact conformation with minimal global rearrangement throughout the simulation. The magnitude of difference between the two RMSD profiles is considerable. While the reference complex approaches fluctuation levels of 0.35 nm, DB00169 remains tightly bounded around ∼0.18 nm for most of the trajectory, reflecting enhanced conformational rigidity and superior complex stability. Taken together, the RMSD analysis indicates that DB00169 confers a markedly more stable interaction with TEAD4 compared to IAG933, with the protein exhibiting reduced backbone mobility and improved structural maintenance over the entire simulation period.

#### 3.8.2 Root mean square fluctuations (RMSF)

The residue-wise flexibility profiles of TEAD4 complexed with IAG933 and DB00169 are shown in Figure 6b. Both complexes displayed similar fluctuation patterns throughout the simulation, indicating that ligand binding did not induce major conformational rearrangements in the receptor. Across the structured core of TEAD4, RMSF values remained low for both complexes, predominantly within 0.04–0.07 nm, consistent with a tightly packed and stable binding environment. Minor differences were evident at a few positions, particularly around residues 236 to 240, where both complexes exhibited a modest increase in mobility, up to ∼0.12 nm for IAG933 and ∼0.13 nm for DB00169. These residues correspond to a surface-exposed region that typically shows enhanced flexibility in TEAD4.

A more prominent peak was observed in both systems around residues 253–256, where RMSF values reached ∼0.20–0.24 nm. This region represents a flexible loop adjacent to the binding pocket, and the comparable magnitude of fluctuation in both complexes suggests that ligand substitution did not substantially alter the natural flexibility of this segment. Beyond residue 300, RMSF values again stabilized between 0.04 and 0.07 nm for both compounds, reflecting a conserved rigidity within the C-terminal domain. The terminal residues displayed mild variability, as expected in disordered regions, but without any marked differences between the two simulations. Overall, the RMSF profiles indicate that both IAG933 and DB00169 maintain the structural integrity of TEAD4, with fluctuations confined to known flexible loops and no significant deviations that would imply destabilization of the protein–ligand complex.

#### 3.8.3 Hydrogen bond analysis

The hydrogen-bonding profiles of the TEAD4 - ligand complexes over the 100 ns trajectory are shown in Figure 6c. Both IAG933 and DB00169 maintained a stable network of interactions with the receptor, consistently forming between one and three hydrogen bonds throughout the simulation. These fluctuations reflect normal dynamic adjustments within the binding pocket and are characteristic of well-stabilized protein - ligand complexes.

The reference complex showed slightly more frequent short-lived hydrogen-bonding transitions, whereas the DB00169 complex exhibited a steadier pattern. However, the overall occupancy patterns for both systems indicate that each ligand sustains reliable polar interactions with TEAD4, without prolonged periods of bond loss or instability. Overall, the hydrogen-bonding results demonstrate that both ligands engage TEAD4 effectively, maintaining continuous polar contacts that contribute to the stability observed in their respective trajectories.

#### 3.8.4 Radius of gyration (Rg)

The compactness of the TEAD4 complexes over the 100 ns simulation was evaluated using Rg, and the profiles are presented in Figure 6d. The Rg trajectory of the reference complex remained largely steady, fluctuating between 1.73 and 1.78 nm, indicating that the protein maintained a well-folded and stable global conformation throughout the simulation. In comparison, the TEAD4 - DB00169 complex exhibited slightly lower and more uniform Rg values, ranging from 1.70 to 1.74 nm, suggesting a marginally tighter packing of the protein in the presence of the hit ligand. This subtle reduction in overall molecular dimensions implies enhanced structural compactness and reduced breathing motions of the protein–ligand assembly. The consistent Rg behaviour across the entire trajectory demonstrates that DB00169 does not induce any destabilizing conformational expansion and instead supports a stable, tightly packed global fold. Such compactness is typically favourable, reflecting improved stabilization of the protein architecture upon ligand binding.

#### 3.8.5 Solvent accessible surface area (SASA)

The dynamic solvent exposure of the complexes was monitored through SASA analysis, illustrated in Figure 6e. The TEAD4 - IAG933 complex displayed SASA values in the range of 124–128 nm^2^, reflecting normal levels of solvent interaction for the protein in its bound state. These fluctuations were modest, confirming that the reference ligand maintains a stable interaction surface without inducing unfolding or significant conformational exposure to the solvent.

The DB00169 complex, however, consistently demonstrated slightly reduced SASA values (121–124 nm^2^). This decrease in solvent-accessible area indicates that DB00169 occupies the TEAD4 binding pocket more effectively, promoting tighter packing and allowing less of the protein surface to remain solvent-exposed. Reduced SASA often corresponds to improved hydrophobic burial and more favourable ligand-induced stabilization. The stable SASA pattern throughout the simulation confirms that DB00169 enhances the structural integrity of the TEAD4 complex without triggering elevated solvent engagement that would be indicative of local unfolding or structural relaxation.

#### 3.8.6 Free energy landscape (FEL)

The conformational energy profiles of the protein–ligand systems were mapped using Gibbs free-energy landscapes, enabling visualization of the thermodynamically preferred states of each complex. The FEL plot of the TEAD4 - IAG933 complex (Figure 6f) revealed a single dominant and well defined energy minima. This compact, unimodal energy well reflects the stabilization of TEAD4 in a largely uniform conformational ensemble, with minimal excursions into higher-energy states. Such a landscape is characteristic of a system that remains locked in a stable conformer throughout the trajectory. In contrast, the DB00169–TEAD4 complex exhibits a more distributed energy landscape with two distinct low-energy minima (Figure 6g), separated by shallow barriers and connected through intermediate green-yellow regions. This bifurcated topology indicates that the complex samples multiple favourable conformational states, reflecting enhanced adaptability of TEAD4 when bound to DB00169.

## 4 Discussion

The present study integrates generative modelling, machine learning based prioritization, and multiscale molecular simulations to identify potential TEAD4 modulators from a limited pool of experimentally validated compounds. By combining GAN-driven descriptor augmentation with structure-based evaluation and molecular dynamics analyses, this workflow enabled systematic refinement of candidate molecules and provided mechanistic insight into TEAD4-ligand recognition. Importantly, this designation is based on comparative computational analyses relative to the reference inhibitor and does not imply confirmed inhibitory activity. The inclusion of the known TEAD4 inhibitor IAG933 throughout the computational workflow provides a form of retrospective validation, enabling comparative assessment of predicted candidates against an established reference.

The integrated structural and dynamic analyses collectively highlight DB00169 as a promising TEAD4-binding candidate within the computational framework. Beyond the favourable conformational dynamics observed for DB00169, its biological relevance further strengthens its potential as a TEAD4-targeting molecule. DB00169 corresponds to cholecalciferol (vitamin D_3_), a compound with well-documented roles in hepatic physiology and disease modulation. Cholecalciferol and its active metabolites have been extensively reported to exert hepatoprotective effects through the suppression of oxidative stress, attenuation of inflammatory signaling, and inhibition of fibrotic progression, pathological processes that are increasingly recognized as being tightly linked to aberrant Hippo pathway activation in liver disease (Guerrero-Guerrero et al., 2025; Han et al., 2013; Iruzubieta et al., 2014).

Vitamin D deficiency has been repeatedly reported in patients with chronic liver diseases and HCC, where reduced circulating levels correlate with disease severity, advanced tumor stage, and poorer clinical outcomes (Markotić et al., 2022; Yi et al., 2021). Mechanistically, vitamin D signaling through the vitamin D receptor (VDR) modulates key pathways involved in hepatic inflammation, immune regulation, and cellular proliferation. Studies have demonstrated that activation of VDR suppresses pro-inflammatory cytokines and profibrotic mediators, thereby mitigating the chronic inflammatory microenvironment that predisposes hepatocytes to malignant transformation (Iruzubieta et al., 2014; Negri et al., 2020). Importantly, recent transcriptomic and systems-level analyses have revealed that vitamin D-related gene signatures are inversely associated with oncogenic signaling pathways in HCC, including pathways governing proliferation, survival, and metabolic reprogramming (Ravaioli et al., 2022; Wang et al., 2024). These findings suggest that vitamin D signaling may exert tumor-suppressive effects at multiple regulatory levels, extending beyond metabolic homeostasis to transcriptional control mechanisms relevant to cancer progression.

In this context, the interaction profiling performed in the present study provides a structural basis for the observed biological associations. DB00169 was found to engage conserved residues within the TEAD4 binding pocket, including Q269 and surrounding hydrophobic residues that define the ligand-interaction groove. These residues overlap with regions critical for protein–protein interactions involving TEAD transcription factors, implying that ligand occupation at this site could sterically or allosterically impair coactivator binding. Such disruption is particularly relevant in HCC, where sustained transcriptional activity downstream of TEAD factors contributes to uncontrolled cell proliferation and survival.

The molecular dynamics simulations further support this interpretation. Compared to the reference compound, DB00169 induced lower RMSD values, a more compact radius of gyration, and stable solvent exposure profiles, collectively indicating enhanced accommodation within the TEAD4 pocket. Hydrogen bond analysis revealed that both complexes maintained comparable interaction counts throughout the simulation, suggesting that DB00169 achieves stability not through excessive polar interactions but via a balanced combination of conserved hydrogen bonding and extensive hydrophobic engagement.

Thermodynamically, the Gibbs free-energy landscape analysis revealed distinct conformational behaviour between the two systems. While the reference compound stabilized TEAD4 in a single dominant energy minimum, DB00169 promoted sampling of two closely related low-energy states. Such an energy landscape reflects conformational adaptability rather than instability, allowing the protein–ligand complex to explore multiple favourable configurations without accessing high-energy conformers. This property may be particularly advantageous for modulating transcription factor function, where subtle shifts in protein dynamics can significantly influence downstream signaling outcomes.

Beyond its direct interaction with TEAD4, the established biological profile of cholecalciferol provides additional biological context supporting the relevance of DB00169 in hepatic disease pathways. Multiple studies have demonstrated that vitamin D supplementation improves liver function parameters, reduces hepatic inflammation, and attenuates disease progression in conditions such as non-alcoholic fatty liver disease and cirrhosis, both recognized risk factors for HCC development (Erbaş et al., 2014; Mohamed et al., 2023). Additionally, vitamin D has been reported to suppress proliferation and promote differentiation in hepatic cancer cell models, supporting its potential role in limiting tumor growth (Adelani et al., 2021; Yi et al., 2021).

Taken together, the present findings suggest that DB00169 represents a computationally prioritized candidate with potential relevance to TEAD4 binding, supported by its favourable structural and dynamic characteristics. Rather than functioning as a purely synthetic inhibitor, DB00169 bridges molecular recognition with physiological relevance, reinforcing the value of integrative computational pipelines for identifying clinically meaningful candidates. These results warrant further experimental validation to elucidate the extent to which cholecalciferol-mediated TEAD4 modulation contributes to its protective effects in HCC.

While the present study provides multiple layers of computational evidence supporting the interaction of DB00169 with TEAD4, these findings should be interpreted within the limitations of *in silico* methodologies. The predicted binding behaviour, stability, and electronic properties do not constitute direct evidence of biological activity or TEAD4 inhibition. Therefore, DB00169 should be considered a computationally prioritized candidate, and experimental validation through biochemical and cellular assays is necessary to confirm its functional relevance. Future work will focus on validating the predicted candidates through *in vitro* binding assays, TEAD4 functional inhibition studies, and hepatocellular carcinoma cell-based models to establish their biological activity and therapeutic relevance. Accordingly, all computational findings presented here should be interpreted as supportive evidence for candidate prioritization rather than confirmation of direct biological activity.

It is important to note that the novelty of the present study does not lie in the development of a new generative algorithm, but in the integration of descriptor-level generative modelling with downstream predictive and structural analyses within a unified framework. By applying this strategy to TEAD4, a target with limited experimentally validated modulators, this work demonstrates how established computational techniques can be effectively combined to address data scarcity and enable biologically meaningful candidate prioritization.

## 5 Conclusion

This study presents an integrative computational framework for identifying potential TEAD4 modulators relevant to liver diseases by combining descriptor-guided generative modelling with structure- and quantum-based analyses. The use of a conditional GAN enabled effective augmentation of chemically realistic molecules within the QikProp descriptor space, facilitating downstream screening beyond conventional dataset limitations. DB00169 emerged as a prominent candidate, exhibiting favourable predicted binding free energies, stable interaction profiles, and enhanced conformational adaptability within the TEAD4 binding pocket. Molecular dynamics simulations further supported its stability through reduced structural fluctuations and energetically favourable free-energy landscapes compared with the reference compound. Notably, the identification of DB00169 aligns with existing evidence supporting the hepatoprotective relevance of vitamin D signaling. Overall, this work demonstrates the utility of deep generative modelling integrated with molecular dynamics and quantum-informed analyses to uncover biologically meaningful modulators of transcription factor activity. The proposed framework provides a scalable and interpretable strategy for prioritizing candidate molecules for TEAD4-targeted drug discovery, with the identified hits serving as hypotheses for subsequent experimental validation rather than definitive therapeutic leads.

## Data Availability

The data and code supporting the conclusions of this study are publicly available in a GitHub repository: https://github.com/vitmilab/GAN-enabled-molecule-generation.
